# ZnO nanoparticles supported on dendritic fibrous nanosilica as efficient catalysts for the one-pot synthesis of quinazoline-2,4(1*H*,3*H*)-diones†

**DOI:** 10.1039/d1ra07197a

**Published:** 2021-11-17

**Authors:** Farzaneh Shamsa, Alireza Motavalizadehkakhky, Rahele Zhiani, Jamshid Mehrzad, Malihe Sadat Hosseiny

**Affiliations:** Department of Chemistry, Neyshabur Branch, Islamic Azad University Neyshabur Iran amotavalizadeh@yahoo.com R_zhiani2006@yahoo.com; Advanced Research Center for Chemistry Biochemistry & Nanomaterial, Neyshabur Branch, Islamic Azad University Neyshabur Iran; New Materials Technology and Processing Research Center, Neyshabur Branch, Islamic Azad University Neyshabur Iran; Department of Biochemistry, Neyshabur Branch, Islamic Azad University Neyshabur Iran

## Abstract

The transmutation of waste into valuable materials has a special place in green chemistry. Herein, we report the preparation of quinazoline-2,4(1*H*,3*H*)-diones from 2-iodoaniline, isocyanides, and carbon dioxide in the presence of ZnO NPs stably placed on the surface of dendritic fibrous nanosilica by cellulose (DFNS/cellulose-ZnO) as a catalyst. This is a great economic strategy to create three bonds in a one-pot multicomponent reaction step employing functional groups. To prepare the catalyst, the dendritic fibrous nanosilica surface was first activated using cellulose as a substrate to support ZnO NPs. Cellulose acts as a stabilizing and reducing agent for the ZnO nanocatalyst and eliminates the need for a reducing agent. The structure of the prepared DFNS/cellulose-ZnO was examined by various methods, including thermogravimetric analysis (TGA), X-ray diffraction (XRD), scanning electron microscopy (SEM), transmission electron microscopy (TEM), X-ray photoelectron spectroscopy (XPS), and inductively coupled plasma (ICP). The largest amount of quinazoline-2,4(1*H*,3*H*)-diones was obtained under ideal situations in the presence of 5 mg of DFNS/cellulose-ZnO under carbon dioxide (1 atm) utilizing a balloon set at 70 °C for 3 hours. The substance was reused for ten consecutive runs and the quinazoline-2,4(1*H*,3*H*)-dione content was more than 92% each time. This indicates the potential for application in the green and economic production of quinazoline-2,4(1*H*,3*H*)-diones, especially from low-cost feedstocks.

## Abstract 2

The transmutation of waste into valuable materials has a special place in green chemistry.

## Graphical abstract

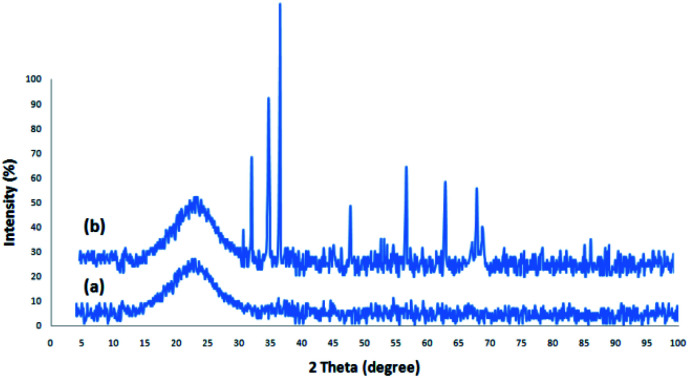

## 1. Introduction

Recently, scientific circles have shown great interest in the employment of non-venomous, recyclable, available, and cost-effective CO_2_ to produce valuable organic composites.^1–4^ Remarkable progress has been made in the area, mostly by d-block metal-catalyzed reactions. However, the preparation of heterocyclic composites like phenyl-annulated heteroarenes *via* the introduction of CO_2_ has been less investigated.^5–11^

Isocyanides have been mainly employed as significant synthons in modern synthetic organic chemistry, especially for N-containing heterocyclic composite synthesis through chemical transformations like multi-component reactions.^12–22^ Today, different transition metal (such as Cu,^24^ Pd,^23^ Ni,^27^ Ag,^25^ and Co^26^)-catalyzed reactions have been reported, in which isocyanides were deployed to generate N-containing heterocyclic molecules. Quinazoline-2,4(1*H*,3*H*)-diones are key structural sub-moieties of a wide range of commercial medicines as well as biological molecules. They are the main framework of anti-hypertensive medicines such as cloperidone,^28^ pelanserin,^29^ ketanserin^30^ as well as anti-psychotic medicines like tioperidone^31^ and belaperidone.^32^ Moreover, these composites are the key mediators for the creation of bio-active *N*3-substituted quinazoline-4(3*H*)-ones and *N*1,*N*3-disubstituted quinazoline-2,4(1*H*,3*H*)-diones.^33–35^

Combining isocyanide and CO_2_ through a multi-element reaction to produce *N*3-substituted 2,4(1*H*,3*H*)-quinazolinediones may seem like an interesting approach. However, in the presence of transition metal catalysts, this sort of multi-element reaction is yet underdeveloped.^34^ The reason is that the solubility of carbon dioxide in organic solvents is very low, and this low solubility is reduced with increasing temperature. Furthermore, isocyanides are solids/liquids that are entirely solvable in organic solvents. Hence, combining CO_2_ and isocyanides in a multi-element reaction is not easy.

Significant efforts have been made to synthesize cyclic carbonates from CO_2_ cycloaddition into oxiranes. Currently, a variety of reusable bifunctional Co(ii), Pb(ii), Zn(ii), Ni(ii), and Cu(ii) compounds have been synthesized to act as effective catalysts without any solvent.^35^ The North group employed chromium and aluminum salphen compounds with TBAB to produce cyclic carbonates.^36^ Bimetallic Co(ii), Cu(ii), and Ni(ii) compounds combined with TBAI for the CO_2_ coupling under moderate reaction situations were announced*.*^37^ The salen-type aluminum compounds synthesized as effective catalysts along with TBAB as co-catalyst under air pressure.^38^ Dinuclear Fe(iii) thioether-triphenolate^39^ and Bi(iii) porphyrin compounds^40^ combined with TBAI have shown superb success in the synthesis of products under atmospheric CO_2_ pressure.

ZnO is a noteworthy photoelectric semiconductor material since its electron transfer rate is very high and it can be conveniently created in a variety of nanostructures.^41^ One-dimensional (1D) zinc oxide nanoparticles (nanotubes, nanowires, and nanorods) are important since they help to provide a direct electrical pathway for electron transfer. This pathway can reduce the recombination of carriers, and transfer photo-created electrons.^42^ Nevertheless, one of the most important disadvantages of ZnO-based photoelectrodes is that the UV fraction can be absorbed due to the large gaps between the ZnO bands, restricting its function.^43^ In order to settle the issue, efforts have been made to develop the absorption spectrum of zinc oxide, including connection with narrow bandgap semiconductors, and elemental doping.^44^ Among the different alternatives to electrocatalytic agents, ZnO has been considered a remarkable nominee for use in the OER on account of its high electrocatalytic efficiency, high chemical stability, and ability to absorb solar radiation.^45–49^ Due to the broad bandgap, this material is employed as a photocatalyst and photoanode under UV light. The use of zinc oxide in the visible light region has been explained by many scientists.^50,51^

**Scheme 1:**
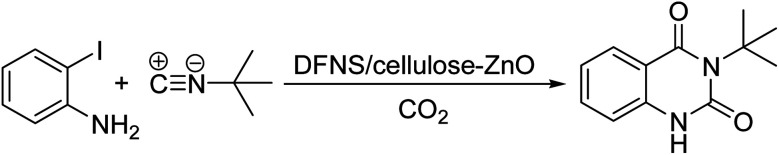
The importance and preparation of quinazoline-2,4(1*H*,3*H*)-diones.

In the view of the above, we decided to provide a heterogeneous catalyst with immobilized ZnO NPs stably placed on the surface of dendritic fibrous nanosilica (DFNS) by cellulose, and examine its catalytic attributes in an efficient three-component reaction to create valuable 2,4(1*H*,3*H*)-quinazolinediones through the deployment of atmospheric carbon dioxide (Scheme 1).

## 2. Experimental section

### 2.1. Materials and methods

Chemical materials were purchased from Fluka and Merck in high purity. Melting points were determined in open capillaries using an Electrothermal 9100 apparatus and were uncorrected. FTIR spectra were recorded on a VERTEX 70 spectrometer (Bruker) in the transmission mode in spectroscopic grade KBr pellets for all the powders. The particle size and structure of the nanoparticles were observed by using a Philips CM10 transmission electron microscope operating at 100 kV. Powder X-ray diffraction data were obtained using a Bruker D8 Advance model with Cu Kα radiation. Thermogravimetric analysis (TGA) was carried out on a NETZSCH STA449F3 at a heating rate of 10 °C min^−1^ under nitrogen. ^1^H and ^13^C NMR spectra were recorded on a BRUKER DRX-300 AVANCE spectrometer at 300.13 and 75.46 MHz, and a BRUKER DRX-400 AVANCE spectrometer at 400.22 and 100.63 MHz, respectively. Elemental analyses for C, H, and N were performed using a Heraeus CHN-O-Rapid analyzer. The purity determination of the products and reaction monitoring were accomplished by TLC on silica gel polygram SILG/UV 254 plates. Mass spectra were recorded on a Shimadzu GCMS-QP5050 Mass Spectrometer.

### 2.2. Synthesis of DFNS@N_3_

DFNS (100 mg) and 3-chloropropyltriethoxysilane (3-CPTES, 5 mL) in 30 mL of ethanol were mixed under nitrogen gas and refluxed for 24 hours. The achieved DFNS@3-chloropropyl was magnetically separated from the blend, washed three times with ethanol, and vacuum-dried at 80 °C for 6 hours. DFNS@3-chloropropyl (1.1 g) was ultrasonically dispersed in a solution of NaN_3_ (1.8 g) in dimethylformamide (50 mL) and mixed at 100 °C for 8 hours. The final solid was gathered magnetically, washed with ethanol, and dried at 100 °C for 10 hours to prepare DFNS@N_3_.

### 2.3. Preparation of alkyne-functionalized cellulose

Cellulose (800 mg) was suspended in NaOH aqueous solution (1.3 wt%) (80 mL) at r.t. for 50 min to swell sufficiently and increase the access of –OH to chemical reagents. The final solid was heated to 50 °C for 40 min and propargyl bromide was released. Then, the blend was mixed at 65 °C for 24 hours, filtered, and rinsed with water and methanol. After drying at 80 °C, alkyne-functionalized cellulose was achieved.

### 2.4. Synthesis of DFNS/cellulose

A blend of alkyne-functionalized cellulose (1600 mg), DFNS@N_3_ (800 mg), sodium ascorbate (2500 mg), and copper(ii) sulfate (100 mg) in 40 mL of dimethylformamide was mixed at 60 °C for 21 hours. Next, the blend was chilled and the product was separated. It was rinsed with DMF and deionized water before vacuum drying at 90 °C for 11 hours.

### 2.5. Synthesis of DFNS/cellulose-ZnO

Zn(CH_3_COO)_2_·2H_2_O (15 mM, 1.9 mL) was poured into a compound of 800 mg DFNS@CL in 45 mL of methanol and ultrasonicated for 0.5 hours. It was then mixed for 22 hours at room temperature and the black solid was magnetically separated, rinsed with DMF, and dried at 90 °C for 6 hours to obtain DFNS/cellulose-ZnO.

### 2.6. Production of *N*3-substituted 2,4(1*H*,3*H*)-quinazolinediones

A blend of 1 mmol of 2-iodoaniline/derivatives, 1 mmol of tertbutyl isocyanides, 1.2 mmol of 1,8-diazabicyclo[5.4.0]undec-7-ene, and DFNS/cellulose-ZnO (5 mg) was added in 5 mL of anhydrous DMF in a 30 mL RB container. The blend was stirred under carbon dioxide (1 atm) utilizing a balloon set at 70 °C. The progress was traced by thin-layer chromatography. The mixture was chilled and evaporated utilizing a rotary evaporator. The product was purified by a column chromatography system using a heptane/EtOAc (10 : 3).

#### 2.6.1. 3-*tert*-Butylquinazoline-2,4(1*H*,3*H*)-dione

White solid, mp: 189–191 °C (lit.: 168–169 °C),^52^ IR (KBr, *ν*, cm^−1^): 3462, 3067, 2975, 2921, 2849, 1717, 1672,1589, 1435, 1190, 1122, 754, 717, 692, 536. ^1^H NMR (CDCl_3_, 400 MHz) *δ* = 10.01 (br s, 1H), 7.98 (dd, *J* = 7.9, 0.7 Hz, 1H), 7.60–7.54 (m, 1H), 7.14–7.10 (ddt, *J* = 7.7, 7.1, 0.7 Hz, 1H), 6.98–6.95 (m, 1H), 1.78 (s, 9H) ppm. ^13^C NMR (CDCl_3_, 400 MHz) *δ* = 164.2, 153.6, 138.4, 134.8, 128.0, 123.1, 117.4, 113.9, 62.3, 29.0 ppm.

#### 2.6.2. 3-*tert*-Butyl-7-methylquinazoline-2,4(1*H*,3*H*)-dione

White solid, mp: 168–170 °C (lit.: 173–174 °C),^52^ IR (KBr, *ν*, cm^−1^): 3467, 3353, 3197, 2928, 1714, 1642, 1535, 1430, 1288, 1163, 814, 786, 651, 548. ^1^H NMR (CDCl_3_, 400 MHz) *δ* = 9.78 (br s, 1H), 7.90 (d, *J* = 8.1 Hz, 1H), 6.99 (ddd, *J* = 8.1, 1.5, 0.6 Hz, 1H), 6.82–6.79 (m, 1H), 2.38 (s, 3H), 1.82 (s, 9H) ppm. ^13^C NMR (CDCl_3_, 400 MHz) *δ* = 164.2, 153.5, 145.3, 138.7, 127.9, 124.6, 114.9, 114.4, 61.8, 30.4, 21.9 ppm.

#### 2.6.3. 3-*tert*-Butyl-6-methylquinazoline-2,4(1*H*,3*H*)-dione

White solid, mp: 182–184 °C (lit.: 189–190 °C),^52^ (KBr, *ν*, cm^−1^): 3417, 3344, 3206, 2932, 1716, 1640, 1532, 1440, 1288, 1156, 814, 735, 642, 527. ^1^H NMR (CDCl_3_, 400 MHz) *δ* = 10.08 (br s, 1H), 7.79 (s, 1H), 7.40–7.32 (m, 1H), 6.93 (d, *J* = 8.2 Hz, 1H), 2.34 (s, 3H), 1.75 (s, 9H) ppm. ^13^C NMR (CDCl_3_, 400 MHz) *δ* = 164.4, 153.2, 136.2, 135.6, 132.8, 127.9, 116.8, 114.0, 62.3, 30.2, 20.6 ppm.

#### 2.6.4. 3-*tert*-Butyl-7-fluoroquinazoline-2,4(1*H*,3*H*)-dione

White solid, mp: 176–178 °C (lit.: 177–178 °C),^52^ IR (KBr, *ν*, cm^−1^): 3352, 3207, 3129, 3072, 2925, 2856, 1720, 1653, 1622, 1484, 1377, 1295, 1242, 1172, 1123, 858, 771, 664, 489. ^1^H NMR (CDCl_3_, 400 MHz) *δ* = 10.24 (br s, 1H), 8.00 (dd, *J* = 8.8, 5.9 Hz, 1H), 6.89 (td, *J* = 8.6, 2.3 Hz, 1H), 6.71 (dd, *J* = 8.9, 2.3 Hz, 1H), 1.80 (s, 9H) ppm. ^13^C NMR (CDCl_3_, 400 MHz) *δ* = 166.4, 163.2, 153.6, 139.8, 131.5, 113.4, 111.1, 100.9, 62.3, 29.9 ppm.

#### 2.6.5. 3-*tert*-Butyl-6-chloroquinazoline-2,4(1*H*,3*H*)-dione

White solid, mp: > 300 °C (lit.: > 300 °C),^53^ IR (KBr, *ν*, cm^−1^): 3448, 3352, 193, 3076, 2992, 2851, 1723, 1640, 1544, 1468, 1363, 1290, 1234, 820, 774, 712, 648, 511. ^1^H NMR (CDCl_3_, 400 MHz) *δ* = 11.19 (s, 1H), 7.73 (dd, *J* = 2.5, 0.5 Hz, 1H), 7.64 (dd, *J* = 8.7, 2.5 Hz, 1H), 7.12 (dd, *J* = 8.6, 0.5 Hz, 1H), 1.63 (s, 9H) ppm. ^13^C NMR (CDCl_3_, 400 MHz) *δ* = 163.0, 150.6, 137.9, 134.1, 126.7, 125.8, 117.4, 116.2, 60.3, 29.5 ppm.

## 3. Results and discussion

### 3.1. Synthesis of the catalyst

**Scheme 2:**
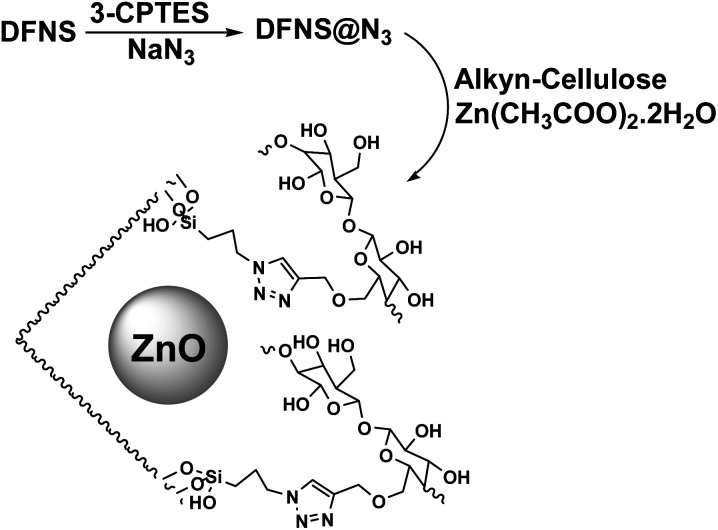
Synthetic pathway for the generation of DFNS/cellulose-ZnO.

The catalyst was synthesized according to Scheme 2. Dendritic fibrous nanosilica was activated by a condensation reaction between its surface hydroxyl groups and 3-CPTES. The structure of DFNS@N_3_ was then produced by replacing the surface of –Cl groups with azide ions. The generated DFNS@N_3_ was then reacted with pre-synthesized alkyne-functionalized cellulose to obtain the DFNS/cellulose by a click reaction. Finally, DFNS@CL was combined with Zn(CH_3_COO)_2_·2H_2_O in absolute ethanol to generate the DFNS/cellulose-ZnO.

### 3.2. Catalytic properties of catalyst

**Fig. 1:**
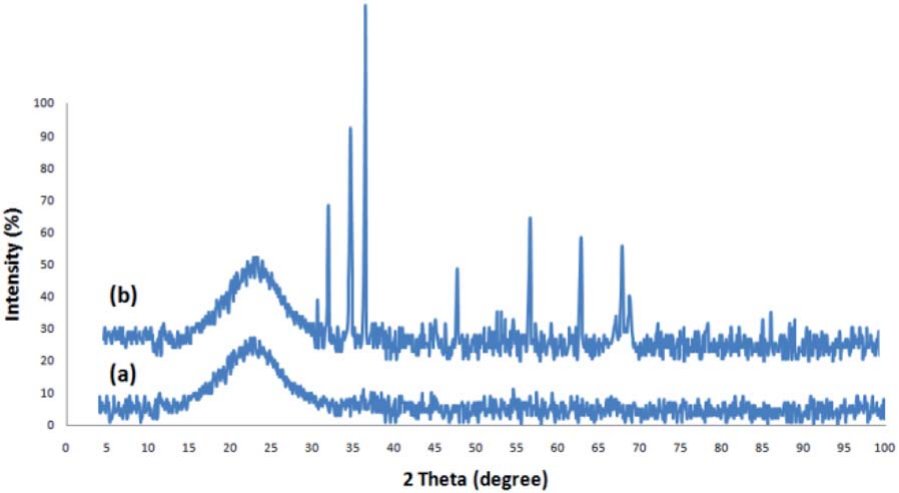
XRD analysis of DFNS (a), and DFNS/cellulose-ZnO (b).

Fig. 1 depicts the XRD design of dendritic fibrous nanosilica and DFNS/cellulose-ZnO. The broad peak in the range of 18–35° belongs to amorphous silica (Fig. 1a). Fig. 1b depicts the XRD design of ZnO catalysts at low and high angles. XRD analysis was employed to investigate the ZnO nanostructures. They were properly matched with pure fluorite ZnO, and there were no impurities. The above designs were in perfect agreement with the ZnO hexagonal structure based on JCPDS card no. 01-089-0510. This confirmed the expansion of ZnO particles on the outer layer of DFNS/cellulose.

**Fig. 2:**
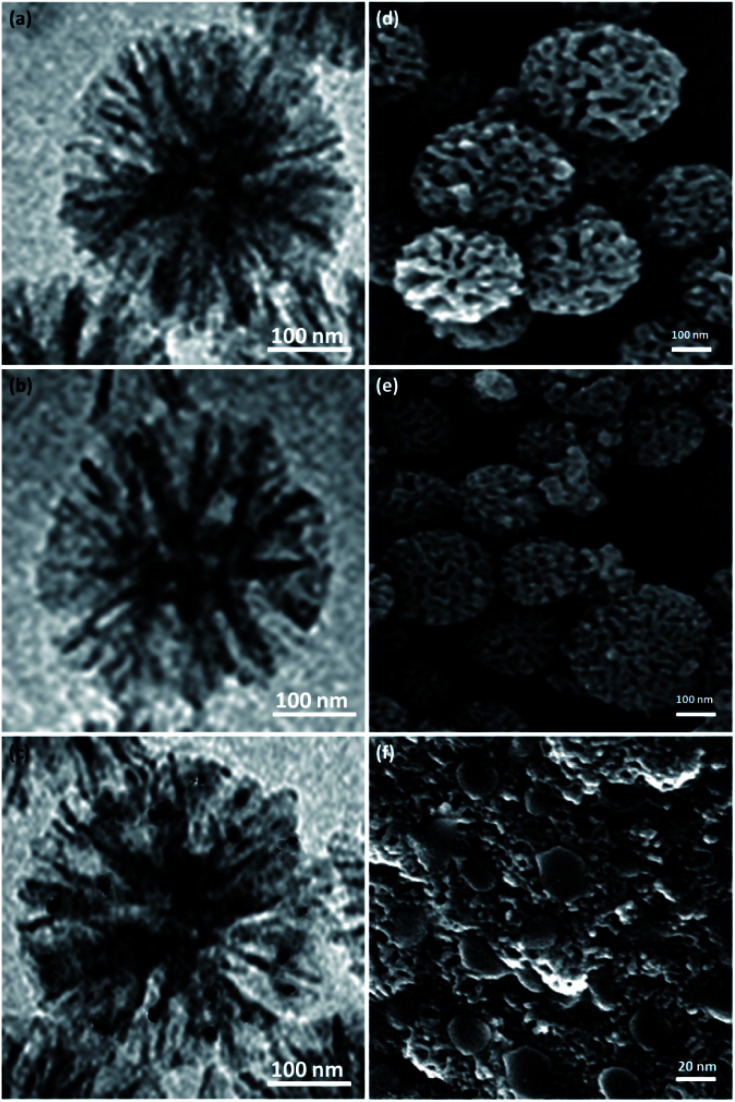
TEM schemas of DFNS (a), DFNS/cellulose (b), DFNS/cellulose-ZnO (c). SEM schemas of DFNS (d), DFNS/cellulose (e), DFNS/cellulose-ZnO (f).

The structures and morphologies of the NPs were investigated by employing SEM and TEM methods. The TEM picture of a DFNS specimen with the radial structure of uniform spheres (350 nm in diameter) and high-quality texture is shown in Fig. 2a. Wrinkled fibers with thicknesses of about 15 nm were observed. The fibers were scattered three-dimensionally from the center after growth. The open pores were generated conically, utilizing radial structures. The SEM picture depicts the solid fibrous essence of the sphere (Fig. 2d). Active surfaces became more available and reactant transfer through the channels was facilitated. The TEM and SEM photos of DFNS/cellulose confirmed that the morphology of dendritic fibrous nanosilica was not altered after modification (Fig. 2b and e). Fig. 2c and 1f display FESEM and TEM photos of the ZnO. As can be seen, spherical metal nanoparticles did not accumulate significantly. ZnO nanoparticles with a diameter of 15–25 nm were attached to the dendritic fibrous nanosilica wall.

**Fig. 3:**
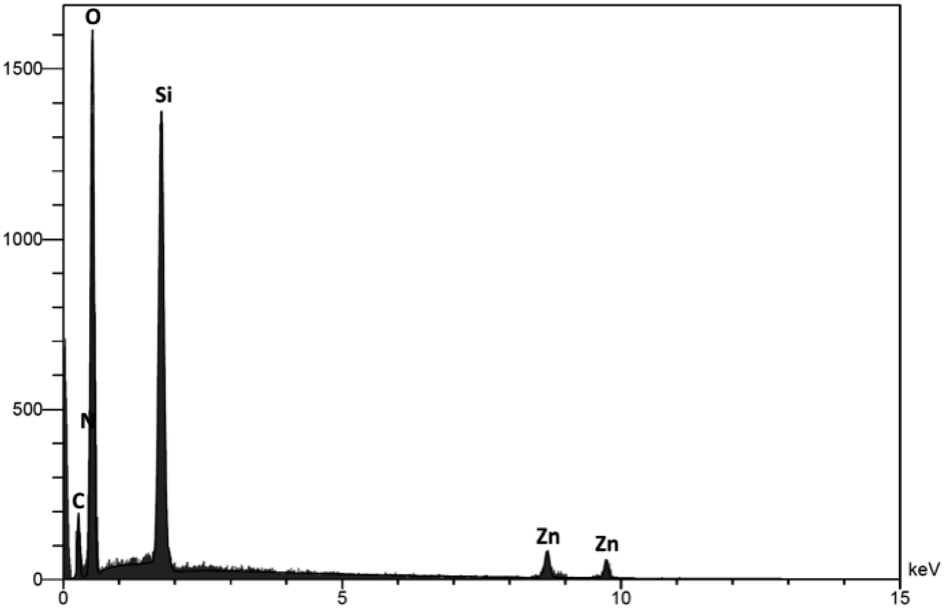
EDS spectra of DFNS/cellulose-ZnO NPs.

EDS analysis was applied to investigate the chemistry of the DFNS/cellulose-ZnO NPs level (Fig. 3). The peaks of Zn, N, O, C, and Si can be seen, and the participation of N confirmed that dendritic fibrous nanosilica was functionalized by employing Cl. The existence of Zn in the catalyst was proved by its corresponding peak.

**Fig. 4:**
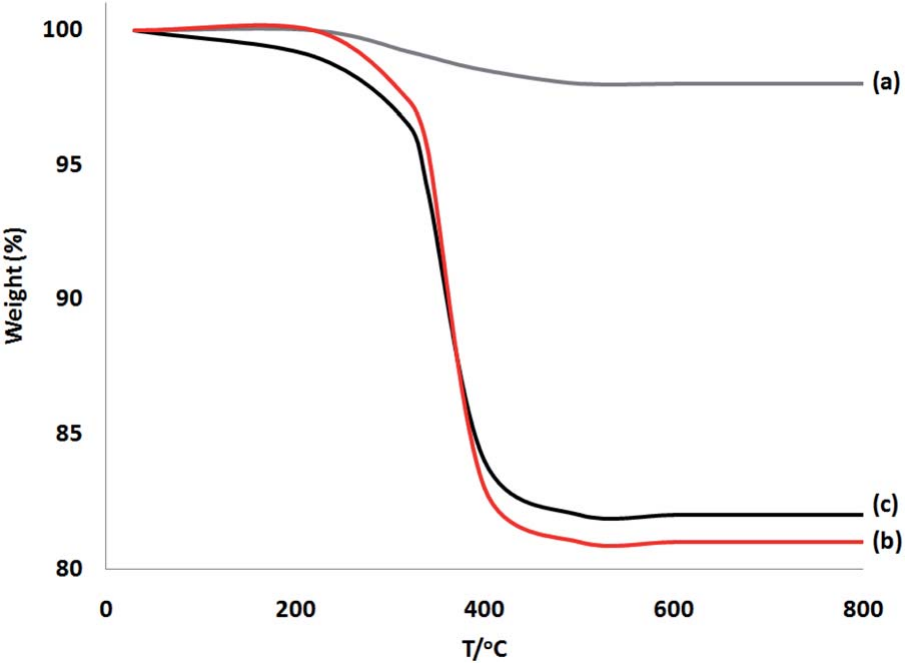
TGA diagram of (a) DFNS, (b) DFNS/cellulose, and (c) DFNS/cellulose-ZnO NPs.

TGA of these materials was performed at a range of temperatures (from room temperature to 750 °C) to prove the thermal stability (Fig. 4). Weight reduction at 190 °C was due to the removal of chemisorbed and physisorbed solvents on the surface of the dendritic fibrous nanosilica. Organic mass reductions (in the range of 220–420 °C) of DFNS/cellulose and DFNS/cellulose-ZnO NPs were 19.1% and 18.3%, respectively. The findings showed that organic structures were supported on the DFNS surface.

### 3.3. Mechanism of the one-pot reaction

**Fig. 5:**
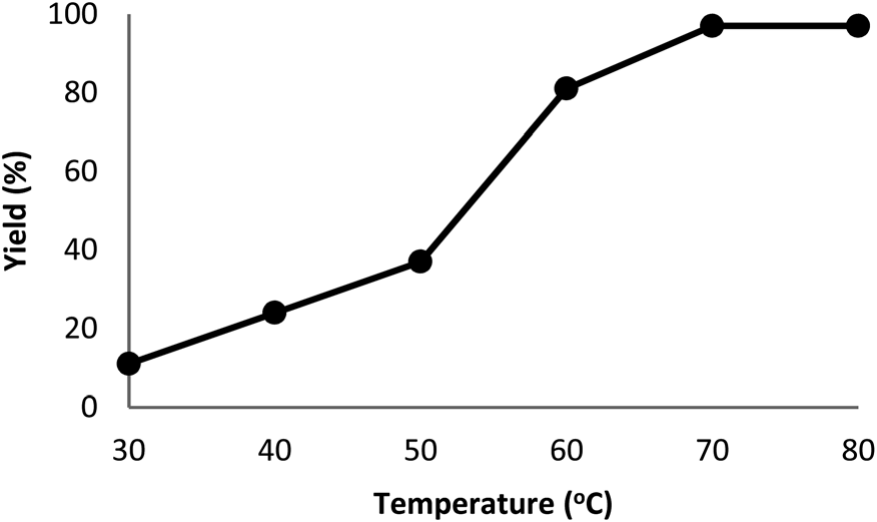
The impact of temperature on the synthesis efficiency of quinazoline-2,4(1*H*,3*H*)-dione.

The DFNS/cellulose-ZnO catalyst was investigated in the binding reaction between CO_2_ insertion into isocyanides and 2-iodoaniline. To determine the best reaction state, the impact of various parameters like solvent, temperature, catalyst quantity, and reaction time on the development of the binding reaction between CO_2_ insertion into isocyanides and 2-iodoaniline was investigated with the catalyst. First, the influence of temperature on the reaction efficiency was examined (Fig. 5). The results showed that the reaction temperature (30–80 °C) and the cyclic carbonate efficiency were directly related. Finally, the quinazoline-2,4(1*H*,3*H*)-dione synthesis reactions were done at 70 °C.

**Fig. 6:**
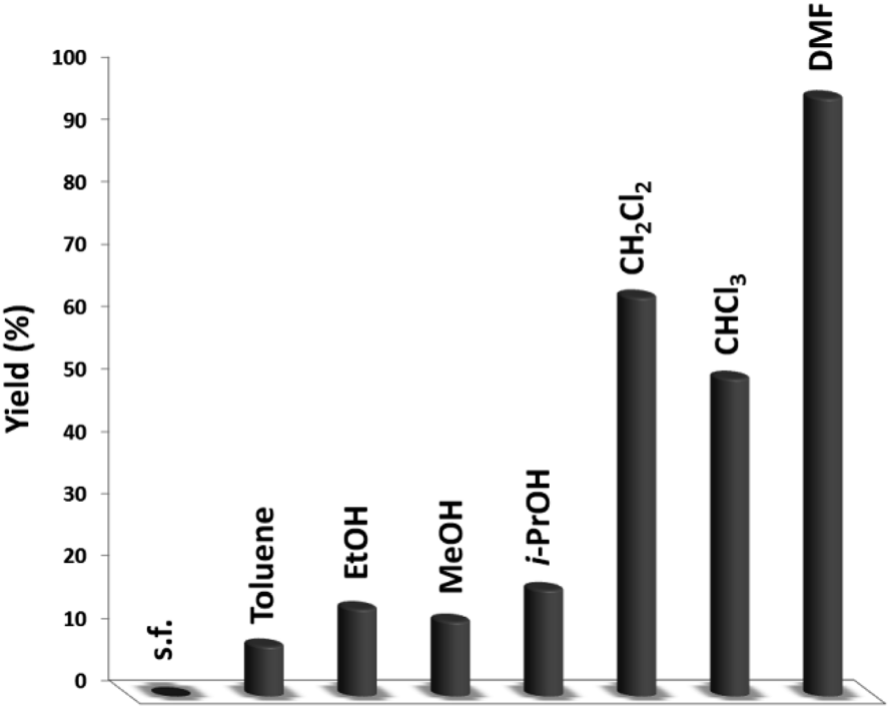
The synthesis of quinazoline-2,4(1*H*,3*H*)-dione in different solvents.

Fig. 6 illustrates the impact of solvent on catalytic progress. The reaction did not begin under toluene or solvent-free situations and demonstrated low conversion in polar protic solvents, including ethanol, methanol, and i-PrOH. CH_2_Cl_2_ and CHCl_3_ resulted in 64% and 51% yields, respectively. The cross-coupling product showed a relatively average-to-good yield with CH_2_Cl_2_ and CHCl_3_ as the polar aprotic solvents.^54^ DMF is considered the finest solvent for reactions and it generated a high efficiency of 96%. The solvent deeply influenced the catalytic activity of the DFNS/cellulose-ZnO NPs towards the synthesis of quinazoline-2,4(1*H*,3*H*)-dione derivatives. Therefore, DMF was selected as a cost-effective solvent.

**Fig. 7:**
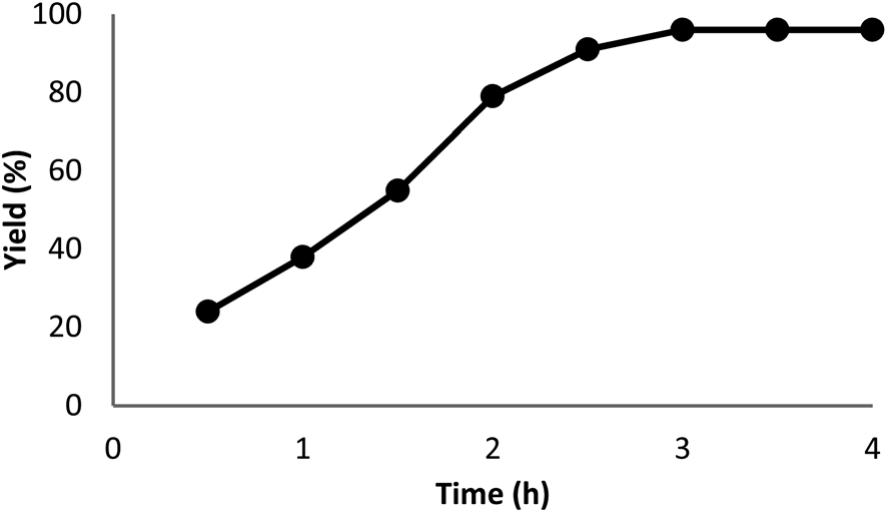
The impact of reaction time on the synthesis function of quinazoline-2,4(1*H*,3*H*)-dione.

**Fig. 8:**
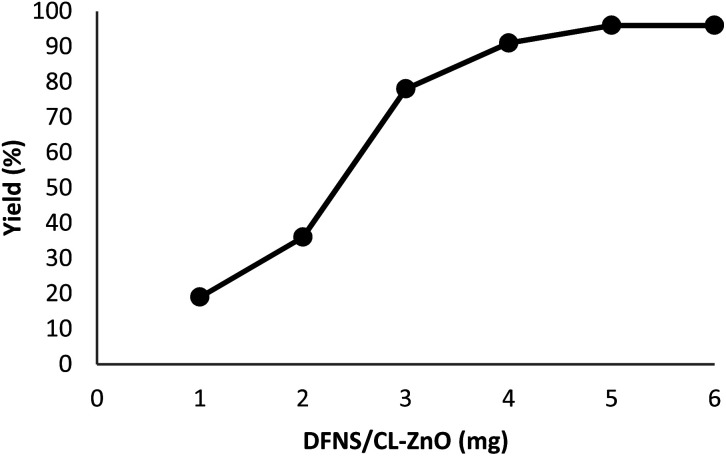
The impact of catalyst quantity on the synthesis performance of quinazoline-2,4(1*H*,3*H*)-dione.

The impact of the reaction duration on the yield of quinazoline-2,4(1*H*,3*H*)-dione under equivalent reaction situations (at 70 °C and 3 MPa CO_2_) is illustrated in Fig. 7. It was discovered that increasing the reaction time positively affected the product yield. In practice, the binding reaction reached 96% within 3 hours; therefore, 3 hours was determined as the optimal time. Fig. 8 depicts the impact of catalyst quantity on the transformation of raw materials into products. Although increasing the catalyst quantity increased the yield percentage, this increase was not significant. Therefore, the finest catalyst value of 5 mg was determined.

**Fig. 9:**
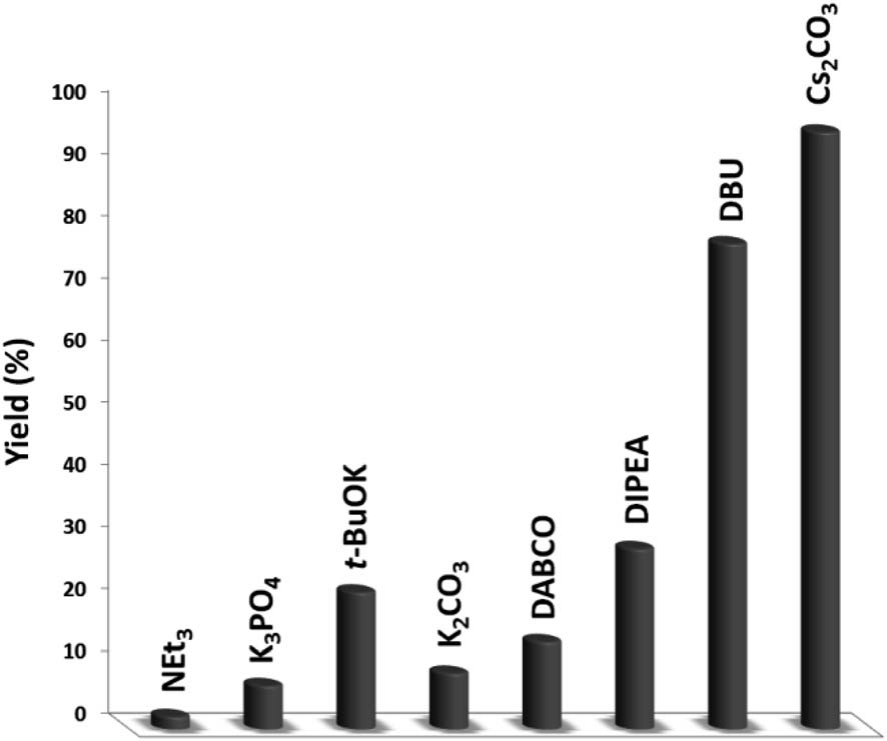
The impact of the base on the synthesis of quinazoline-2,4(1*H*,3*H*)-dione.

In the next stage, we synthesized quinazoline-2,4(1*H*,3*H*)-dione with a variety of bases that were simply isolated from the product (Fig. 9). The baseless synthesis of quinazoline-2,4(1*H*,3*H*)-dione was very weak. The participation of the base in the reaction system remarkably increased the reaction progress. Bases can instigate the reaction, and reducing the quantity of base decreases the product conversion. As expected, the Cs_2_CO_3_ maintained its stability throughout the process, guaranteeing the higher yield of quinazoline-2,4(1*H*,3*H*)-dione.

### 3.4. The efficiency of the system

Under the best reaction situations, we examined the bed range of different derivatives of *o*-iodoanilines (Table 1). The methyl group as an electron-donor group containing 2-iodoanilines provides >90% of the isolated efficiency of the product. When mild electron-withdrawing groups like cyano, chloro, or fluoro substituted *o*-iodoanilines were introduced to the reaction, moderate to very fine isolated yields (81–98%) were achieved. The substituted 2-iodoaniline derivative also generated 86% of the quinazolinedione product yield.

**Table 1:** Synthesis of quinazoline-2,4(1*H*,3*H*)-dione derivatives with the participation of DFNS/cellulose-ZnO NPs^a^

Entry	Substrate	Product	Yield^b^ (%)
1	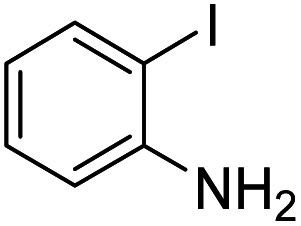	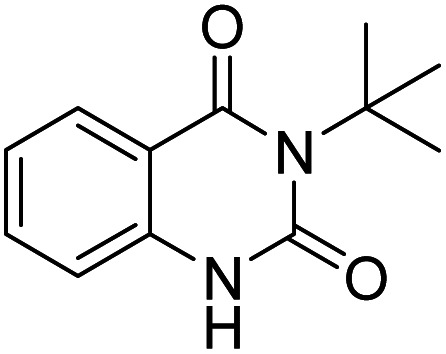	96
2	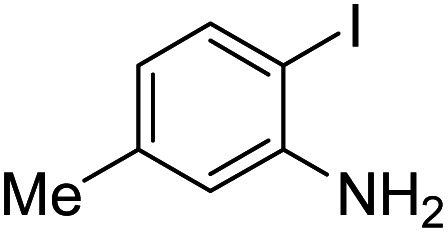	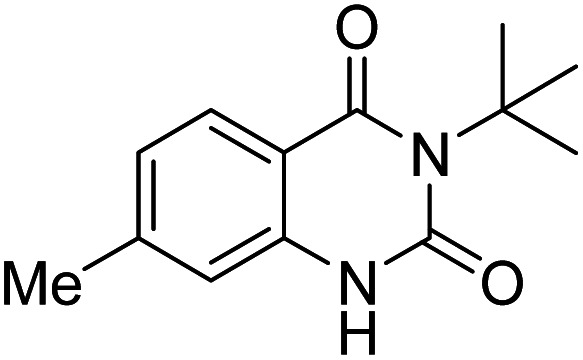	98
3	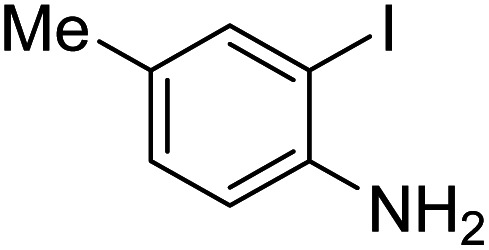	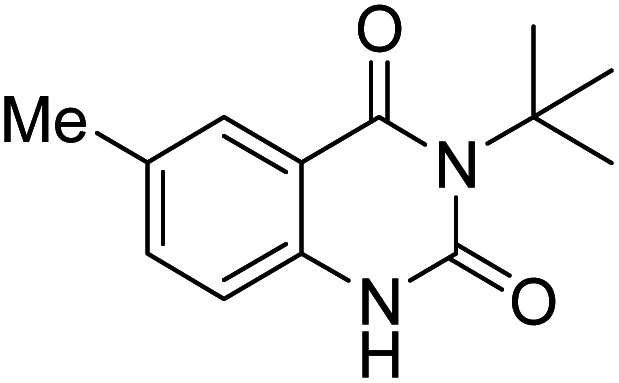	96
4	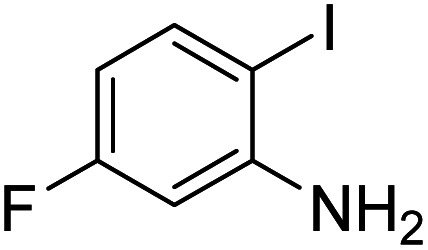	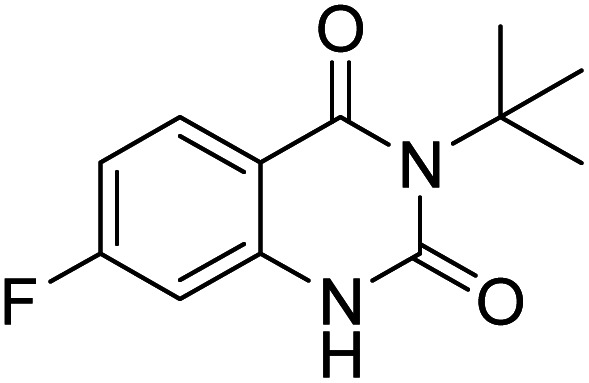	84
5	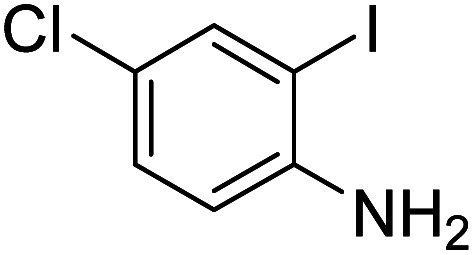	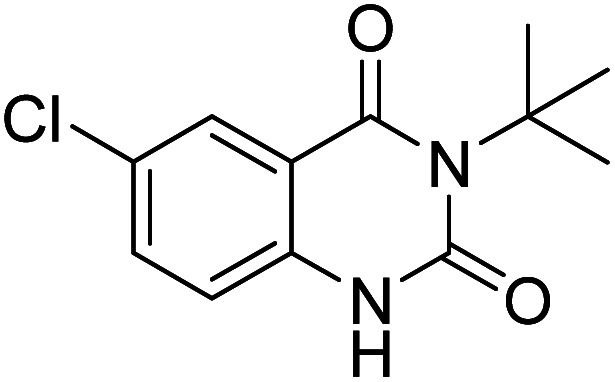	88

a Reaction situations: substrate (1.0 mmol), isocyanide (1.0 mmol), DFNS/cellulose-ZnO (5 mg), Cs_2_CO_3_ (1.0 mmol), DMF (5 mL), 70 °C, CO_2_ balloon, 3 hours.

b Isolated yields.

**Scheme 3:**
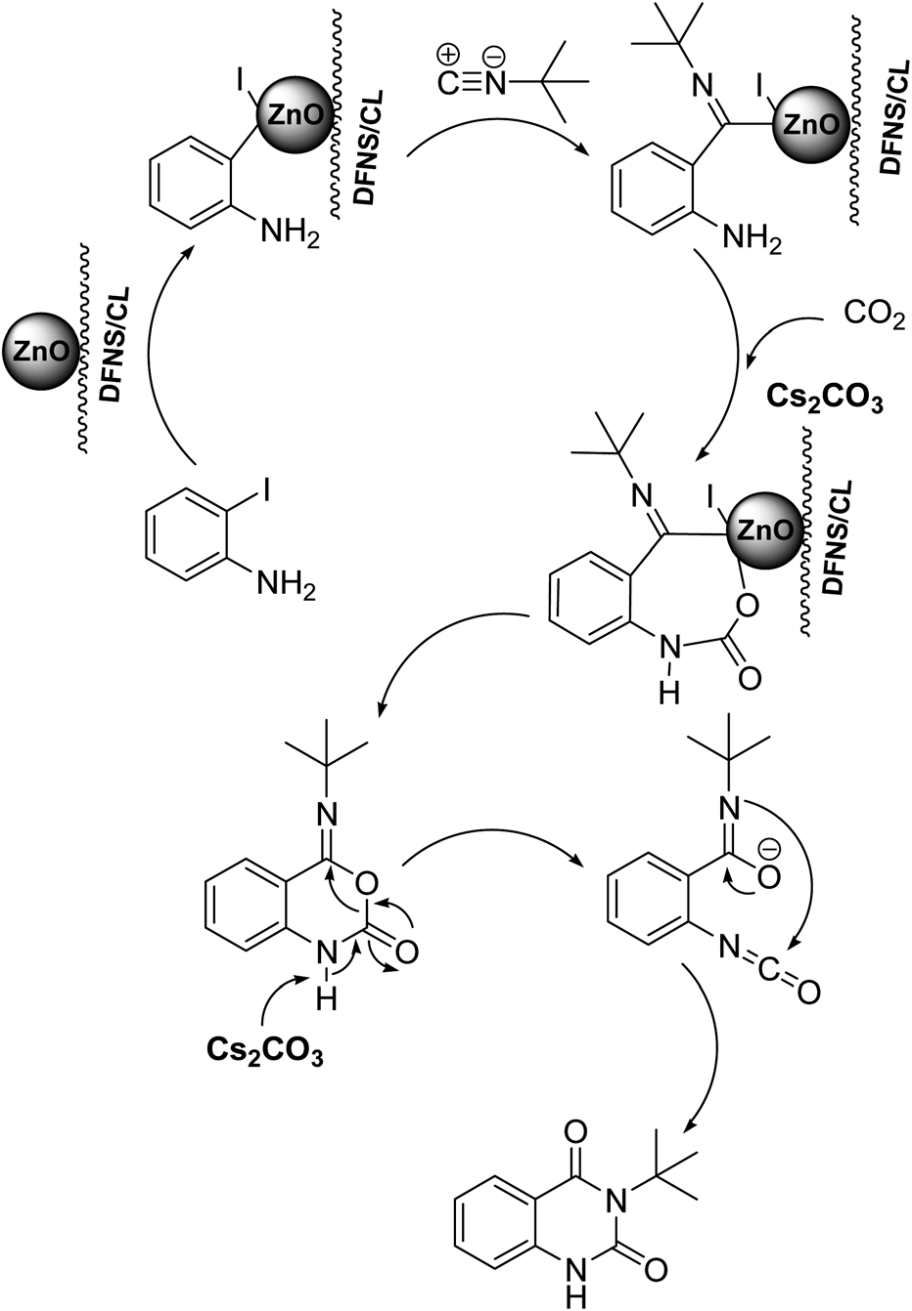
An acceptable mechanical pathway for the generation of 3-(*tert*-butyl)-quinazoline-2,4(1*H*,3*H*)-dione.

To understand the reaction mechanics and the impact of the catalyst, an acceptable mechanism for the *t*-BuNC cycloaddition and *o*-iodoaniline with carbon dioxide in the participation of DFNS/cellulose-ZnO and Cs_2_CO_3_ has been illustrated in Scheme 3. A systematic study showed that *tert*-butyl isocyanide reacted and *o*-iodoaniline did not react with carbon dioxide. Nevertheless, adding *tert*-butyl isocyanide and *o*-iodoaniline promoted the catalysis of cycloaddition with carbon dioxide. The single aniline–catalyst pair moved to the final carbon of carbon dioxide, and ultimately produced the catalytic product (*Z*)-4-(*tert*-butylimino)-1*H*-benzo[*d*][1,3]oxazin-2(4*H*)-one. At the same time, the catalytic product experienced a particular reshuffling in the participation of 1,8-diazabicyclo[5.4.0]undec-7-ene to obtain 3-(*tert*-butyl)quinazoline-2,4(1*H*,3*H*)-dione.

For a more precise assessment of the efficiency of the catalyst, control investigations were conducted (Table 2). The reaction performed with dendritic fibrous nanosilica revealed that no quinazoline-2,4(1*H*,3*H*)-dione was generated after 3 hours (Table 2, entry 1). Moreover, no reaction occurred when DFNS/cellulose-ZnO was utilized as a catalyst (Table 2, entry 2). Cellulose did not have satisfactory catalytic activity. The findings were compared to a variety of other similar catalysts. On account of these dissatisfactory findings, studies were continued to enhance the efficiency by releasing ZnO (Table 2, entry 3). The findings revealed that the synthesis of quinazoline-2,4(1*H*,3*H*)-dione is fundamentally catalyzed by employing ZnO compounds in the DFNS/cellulose. Nanoparticles enhanced the activity of the catalyst on account of the increase in surface area to volume. Therefore, they remarkably enhanced the sensitivity between the catalyst and the reactants and functioned as a homogeneous catalyst (Table 2, entry 3 and entry 4).

**Table 2:** The influence of different catalysts for the preparation of quinazoline-2,4(1*H*,3*H*)-dione^a^

Entry	Catalyst	Yield^b^ (%)
1	DFNS	—
2	DFNS/cellulose	—
3	DFNS/cellulose-ZnO	96
4	Cellulose-ZnO	98

a Reaction conditions: substrate (1.0 mmol), isocyanide (1.0 mmol), catalyst (5 mg), Cs_2_CO_3_ (1.0 mmol), DMF (5 mL), 70 °C, CO_2_ balloon, 3 hours.

b Isolated yield.

### 3.5. The recyclability of the catalyst

ZnO leaching was investigated by inductively coupled plasma mass spectrometry (ICP-MS) analysis of the catalyst, after ten cycles of reactions. The loading quantity of ZnO was 2.0 wt%, which revealed negligible ZnO NPs leaching. The findings proved the high recyclability of the ZnO nanocatalyst. The amount of ZnO loaded on DFNS/ZnO was determined by ICP-MS. The amount of ZnO NPs in DFNS/cellulose-ZnO was almost equal to DFNS/ZnO. Interestingly, after ten consecutive reuses of the catalyst, the amount of ZnO in DFNS/cellulose-ZnO was about twice that of DFNS/ZnO NPs (Table 3). This incredible ability of the DFNS/cellulose-ZnO can be assigned to cellulose units that efficiently control the reaction by preventing ZnO agglomeration, as well as recapturing and releasing ZnO during the reaction process.

**Table 3:** The loading amount of ZnO NPs

Entry	Catalyst	wt%
1	DFNS/ZnO	2.4
2	DFNS/cellulose-ZnO	2.2
3	DFNS/ZnO after ten reuses	1.5
4	DFNS/cellulose-ZnO after ten reuses	2.0

**Fig. 10:**
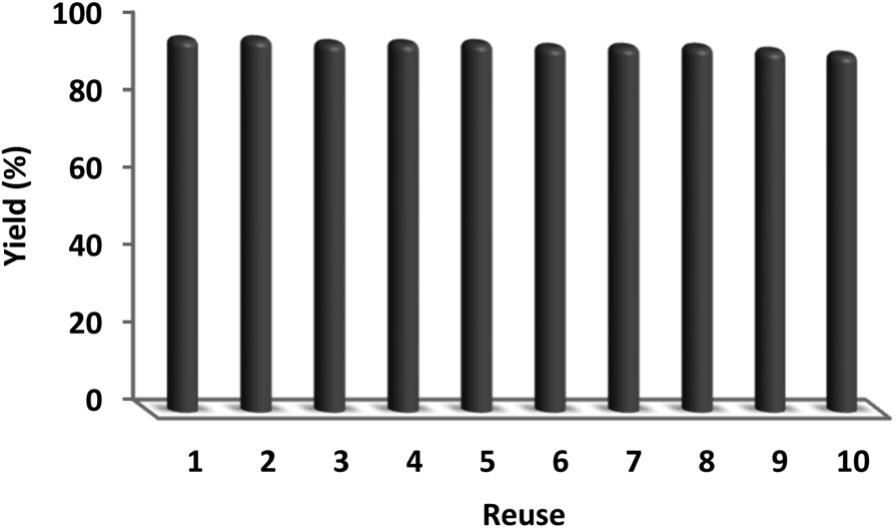
Recyclability of the catalyst.

In sustainable chemistry, catalyst recyclability is considered a remarkable feature. Therefore, the reuse of the DFNS/cellulose-ZnO NPs was examined with respect to the optimal synthesis of quinazoline-2,4(1*H*,3*H*)-dione. DFNS/cellulose-ZnO NPs were conveniently separated from the liquid reaction after a few seconds. The solvent can be employed swiftly after cleaning. According to Fig. 10, the catalyst was reused for ten continuous cycles. Product efficiency was 91% in the tenth run, revealing just a 5% reduction.

**Fig. 11:**
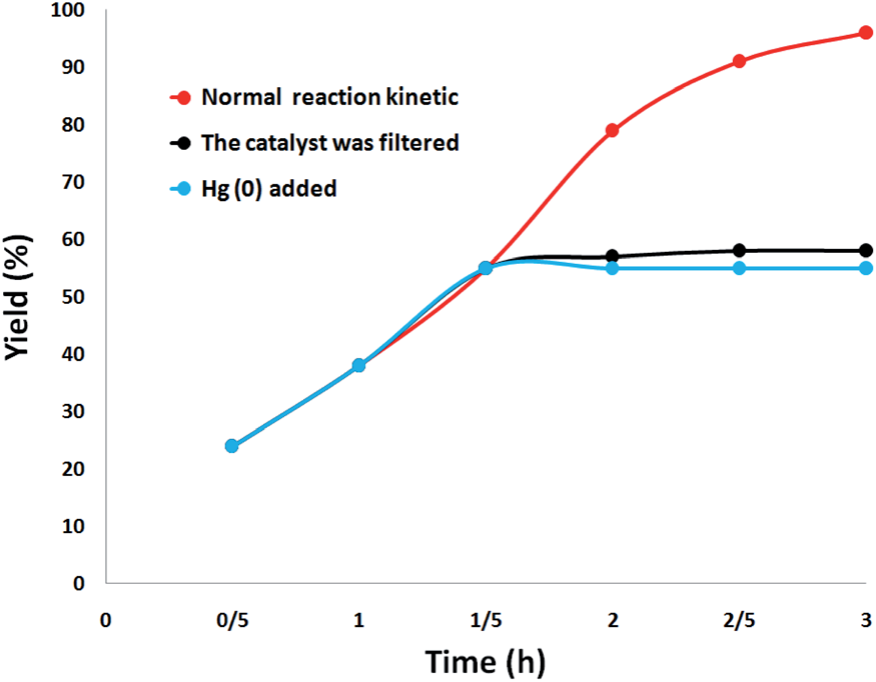
Leaching test for the catalyst in the synthesis of quinazoline-2,4(1*H*,3*H*)-dione.

The heterogeneity of the catalyst was comprehensively studied. First, a hot filtration test was performed for the synthesis of quinazoline-2,4(1*H*,3*H*)-dione under optimal conditions and revealed that DFNS/cellulose-ZnO was removed at a yield of 55% (after 1.5 hours). After the removal of DFNS/cellulose-ZnO, it was revealed that the free catalyst residues were relatively active, and the conversion of 58% was obtained after 3 hours of synthesis of quinazoline-2,4(1*H*,3*H*)-dione. This illustrated the heterogeneity of the catalyst during the reaction and the leaching was partial. Eventually, a mercury-poisoning test was run to investigate the heterogeneity of DFNS/cellulose-ZnO. Mercury (0) remarkably attenuates the metal catalyst on the active exterior and calms its activity; therefore, proving the heterogeneous essence of DFNS/cellulose-ZnO. This test was accomplished in optimal situations. After 1.5 hours, about 300 molar mercury was added and mixed. After 3 hours, the poisoned catalyst was not altered. Fig. 11 illustrates the kinetics of the reaction with the participation of Hg (0). Negative experimental findings revealed that the DFNS/cellulose-ZnO was heterogeneous and there was no remarkable ZnO leaching during the synthesis of quinazoline-2,4(1*H*,3*H*)-dione.

**Fig. 12:**
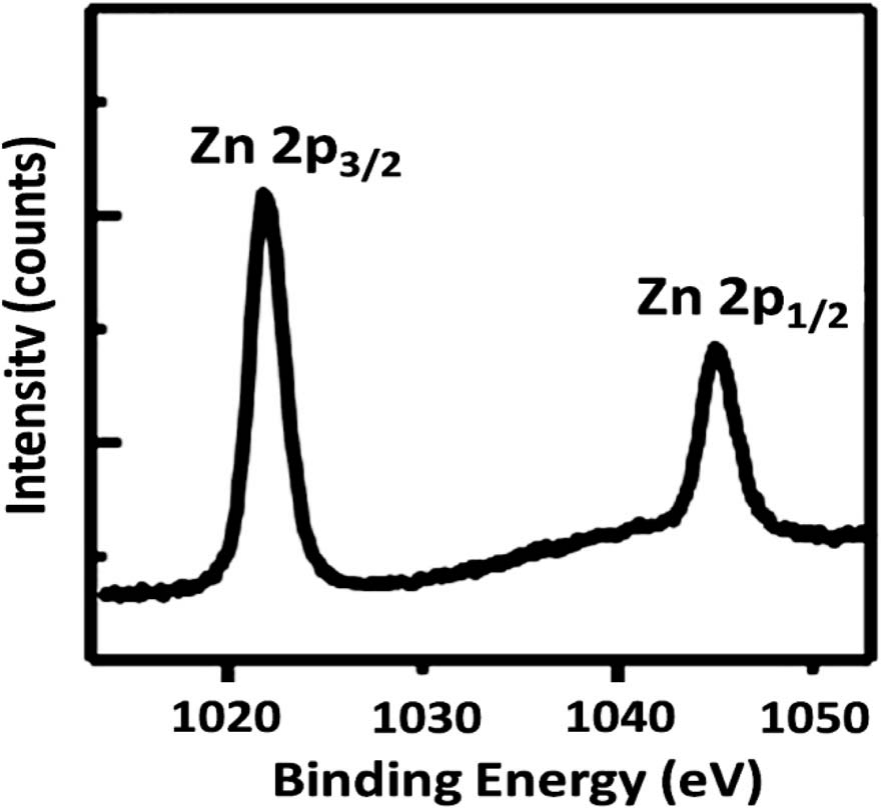
XPS spectra of the DFNS/cellulose-ZnO NPs.

To further understand the underlying reason for the significant difference in recyclability, XPS was employed to characterize the reused DFNS/cellulose-ZnO NPs. The XPS spectra are shown in Fig. 12. For Zn, the Zn 2p_3/2_ and Zn 2p_1/2_ binding energies were determined to be 1045.8 and 1022.8 eV, respectively. Observation of the peaks in regions 1022.8 eV (2p_3/2_) and 1045.8 eV (2p_1/2_) after the catalyst was used ten times proved that Zn NPs were still present in the catalyst structure.

## 4. Conclusions

ZnO nanoparticles were supported on cellulose-modified DFNS through a facile and sustainable reduction technique without any reducing agent to create a separable ZnO catalyst. We have developed the first three-component reaction to synthesize valuable 3-*tert*-butylquinazoline-2,4(1*H*,3*H*)-dione from carbon dioxide, *tert*-butyl isocyanide, and *o*-iodoaniline. Catalyst surface and structure analysis studies by EDX, XPS, TEM, SEM, ICP-MS, TGA, and BET proved the presence of cellulose and the ZnO support on the DFNS surface. This strategy has advantages such as ease of access to raw materials, a wide range of substrates, and good functional group tolerance that allow access to a wide range of 3-*tert*-butylquinazoline-2,4(1*H*,3*H*)-dione derivatives. Therefore, other sustainable NPs with features such as high efficiency and reusability can be developed in the future by following the techniques suggested in this study.

## Conflicts of interest

There are no conflicts to declare.

## Supplementary Material

RA-011-D1RA07197A-s001
