# Supplementary material for: ZnO nanoparticles supported on dendritic fibrous nanosilica as efficient catalysts for the one-pot synthesis of quinazoline-2,4(1H,3H)-diones
Source: RSC Adv. 2021 Nov 17;11(59):37103–11. doi: 10.1039/d1ra07197a (PMC9043541; doi:10.1039/d1ra07197a)
Supplement: RA-011-D1RA07197A-s001 [file RA-011-D1RA07197A-s001.pdf]

Electronic Supplementary Material (ESI) for RSC Advances.
This journal is © The Royal Society of Chemistry 2021

Supporting Information

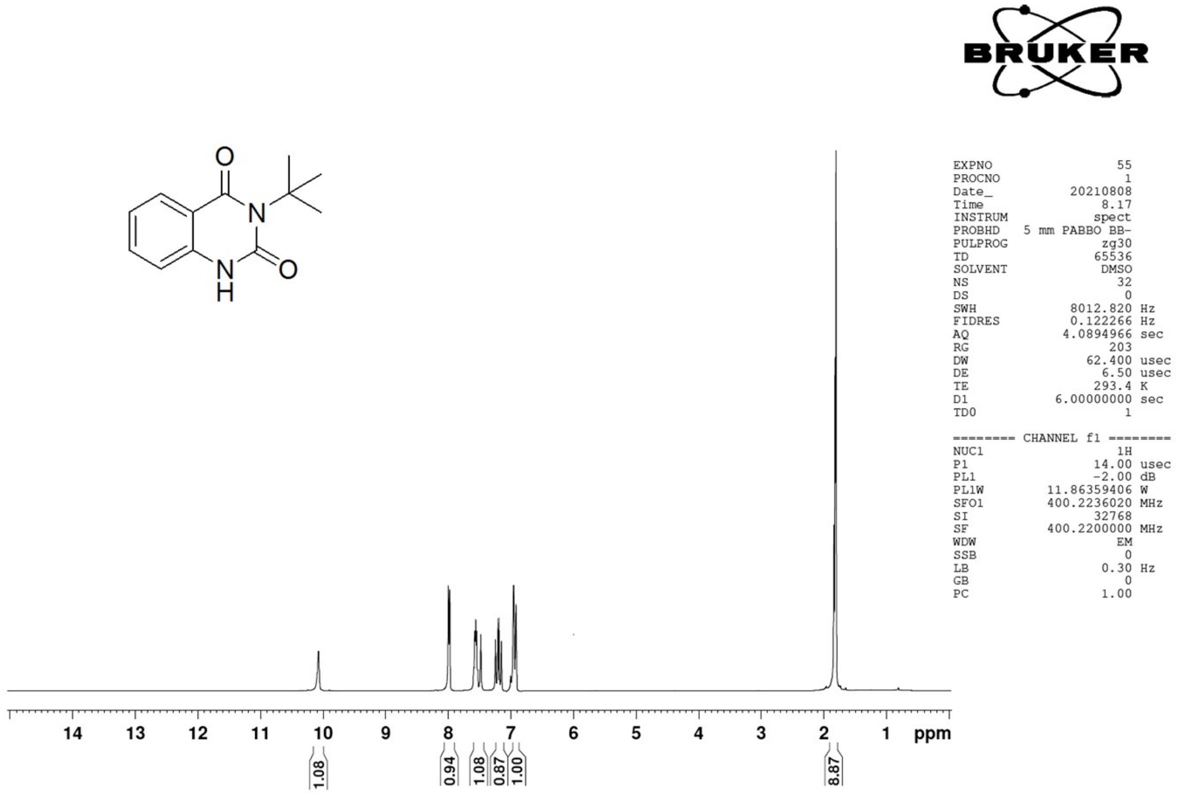

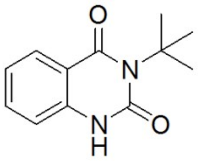

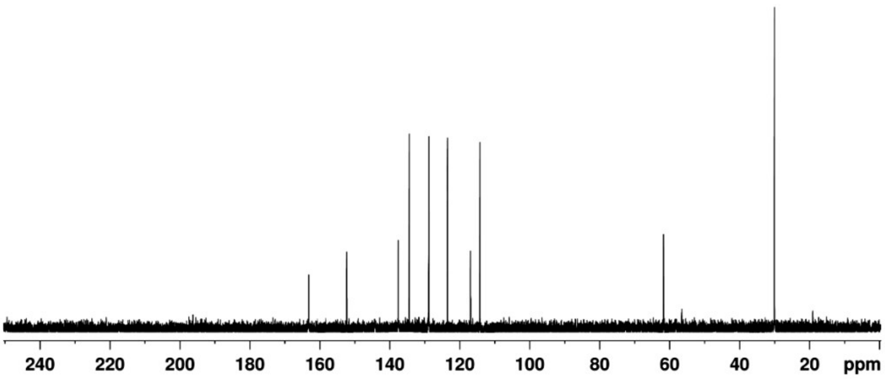


```
EXPNO              138
PROCNO               1
Date_         20210808
Time             18.25
INSTRUM          spect
PROBHD   5 mm PABBO BB-
PULPROG           zgpg
TD               65536
SOLVENT           DMSO
NS                  80
DS                   0
SWH          25252.525 Hz
FIDRES        0.385323 Hz
AQ           1.2976629 sec
RG                2050
DW              19.800 usec
DE                6.50 usec
TE               294.0 K
D1          3.00000000 sec
D11         0.03000000 sec
TD0                  1

======== CHANNEL f1 ========
NUC1               13C
P1                9.00 usec
PL1              -0.90 dB
PL1W       42.02801895 W
SFO1       100.6479784 MHz

======== CHANNEL f2 ========
CPDPRG2        waltz16
NUC2                1H
PCPD2            90.00 usec
PL2              -2.00 dB
PL12             14.16 dB
PL13             17.90 dB
PL2W       11.86359406 W
PL12W       0.28722104 W
PL13W       0.12139934 W
SFO2       400.2216009 MHz
SI               32768
SF         100.6353990 MHz
WDW                 EM
SSB                  0
LB                1.00 Hz
GB                   0
PC                1.40
```

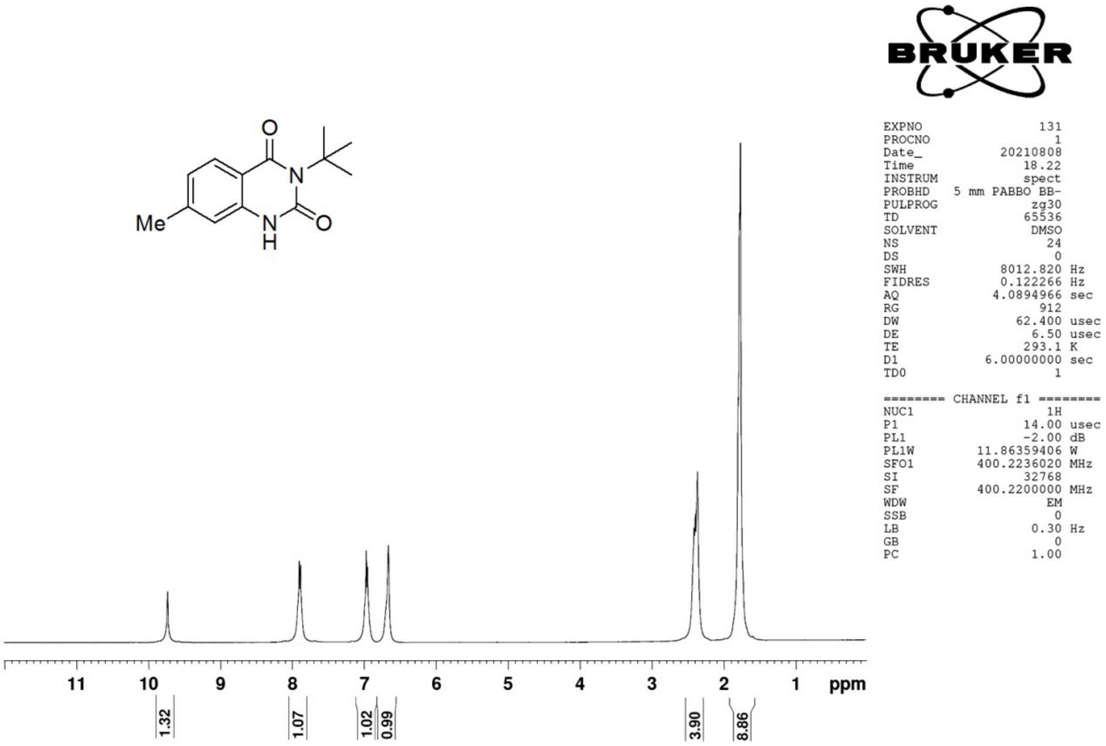

Me
N
H
O
N
O
BRUKER
EXPNO 131
PROCNO 1
Date_ 20210808
Time 18.22
INSTRUM spect
PROBHD 5 mm PABBO BB-
PULPROG zg30
TD 65536
SOLVENT DMSO
NS 24
DS 0
SWH 8012.820 Hz
FIDRES 0.122266 Hz
AQ 4.0894966 sec
RG 912
DW 62.400 usec
DE 6.50 usec
TE 293.1 K
D1 6.00000000 sec
TD0 1
======== CHANNEL f1 ========
NUC1 1H
P1 14.00 usec
PL1 -2.00 dB
PL1W 11.86359406 W
SFO1 400.2236020 MHz
SI 32768
SF 400.2200000 MHz
WDW EM
SSB 0
LB 0.30 Hz
GB 0
PC 1.00
11 10 9 8 7 6 5 4 3 2 1 ppm
1.32
1.07
1.02
0.99
3.90
8.86

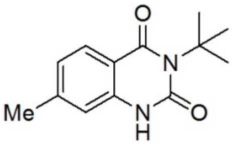

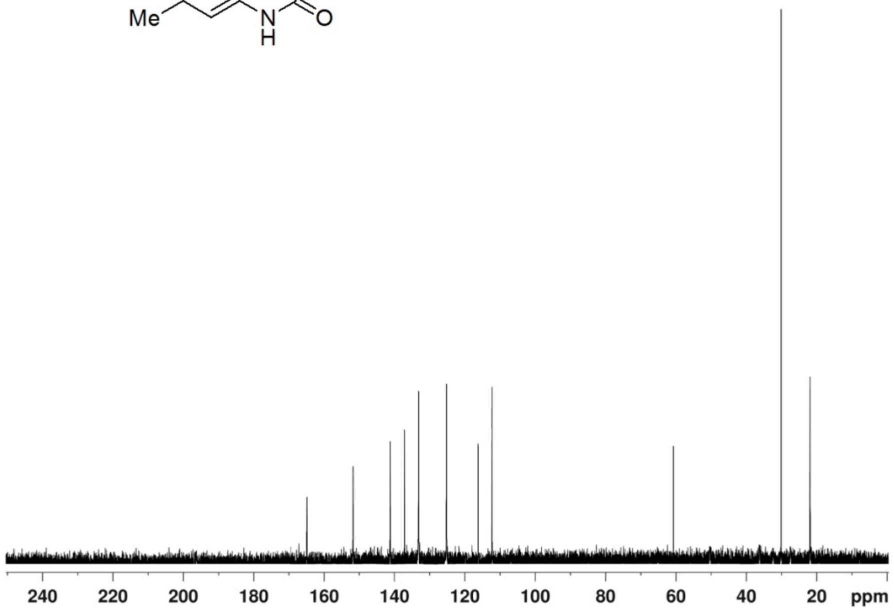


```
EXPNO              134
PROCNO               1
Date_         20210808
Time             17.51
INSTRUM          spect
PROBHD   5 mm PABBO BB-
PULPROG           zgpg
TD               65536
SOLVENT           DMSO
NS                 110
DS                   0
SWH          25252.525 Hz
FIDRES        0.385323 Hz
AQ           1.2976629 sec
RG                2050
DW              19.800 usec
DE                6.50 usec
TE               293.3 K
D1          3.00000000 sec
D11         0.03000000 sec
TD0                  1

======== CHANNEL f1 ========
NUC1               13C
P1                9.00 usec
PL1              -0.90 dB
PL1W       42.02801895 W
SFO1       100.6479784 MHz

======== CHANNEL f2 ========
CPDPRG2        waltz16
NUC2                1H
PCPD2            90.00 usec
PL2              -2.00 dB
PL12             14.16 dB
PL13             17.90 dB
PL2W       11.86359406 W
PL12W       0.28722104 W
PL13W       0.12139934 W
SFO2       400.2216009 MHz
SI               32768
SF         100.6353990 MHz
WDW                 EM
SSB                  0
LB                1.00 Hz
GB                   0
PC                1.40
```

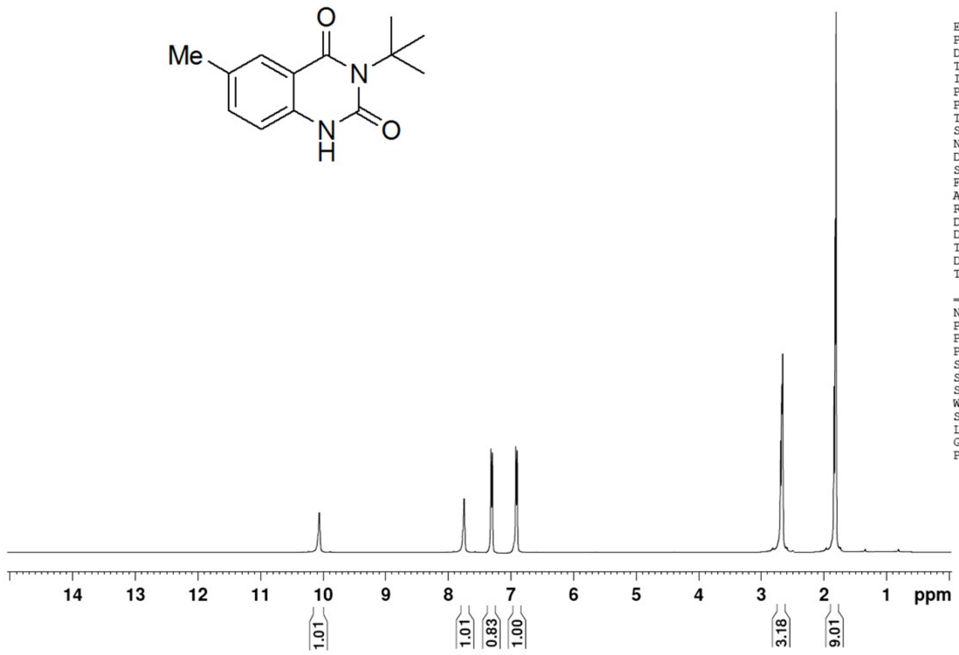


```
EXPNO                 55
PROCNO                 1
Date_           20210808
Time                4.08
INSTRUM            spect
PROBHD   5 mm PABBO BB-
PULPROG             zg30
TD                 65536
SOLVENT             DMSO
NS                    32
DS                     0
SWH             8012.820 Hz
FIDRES          0.122266 Hz
AQ             4.0894966 sec
RG                   203
DW                62.400 usec
DE                  6.50 usec
TE                 293.4 K
D1            6.00000000 sec
TD0                    1

======== CHANNEL f1 ========
NUC1                  1H
P1                 14.00 usec
PL1                -2.00 dB
PL1W         11.86359406 W
SFO1         400.2236020 MHz
SI                 32768
SF           400.2200000 MHz
WDW                   EM
SSB                    0
LB                  0.30 Hz
GB                     0
PC                  1.00
```

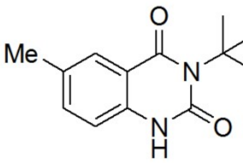

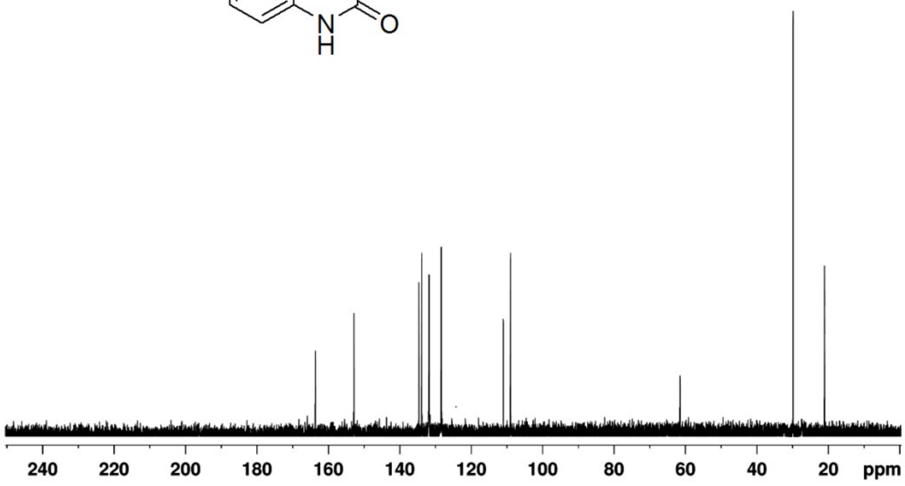


```
EXPNO                  134
PROCNO                   1
Date_             20210808
Time                 17.51
INSTRUM              spect
PROBHD   5 mm PABBO BB-
PULPROG               zgpg
TD                   65536
SOLVENT               DMSO
NS                     110
DS                       0
SWH             25252.525 Hz
FIDRES           0.385323 Hz
AQ              1.2976629 sec
RG                    2050
DW                  19.800 usec
DE                    6.50 usec
TE                   293.3 K
D1             3.00000000 sec
D11            0.03000000 sec
TD0                      1

======== CHANNEL f1 ========
NUC1                   13C
P1                    9.00 usec
PL1                  -0.90 dB
PL1W           42.02801895 W
SFO1           100.6479784 MHz

======== CHANNEL f2 ========
CPDPRG2            waltz16
NUC2                    1H
PCPD2                90.00 usec
PL2                  -2.00 dB
PL12                 14.16 dB
PL13                 17.90 dB
PL2W           11.86359406 W
PL12W           0.28722104 W
PL13W           0.12139934 W
SFO2           400.2216009 MHz
SI                   32768
SF             100.6353990 MHz
WDW                     EM
SSB                      0
LB                    1.00 Hz
GB                       0
PC                    1.40
```

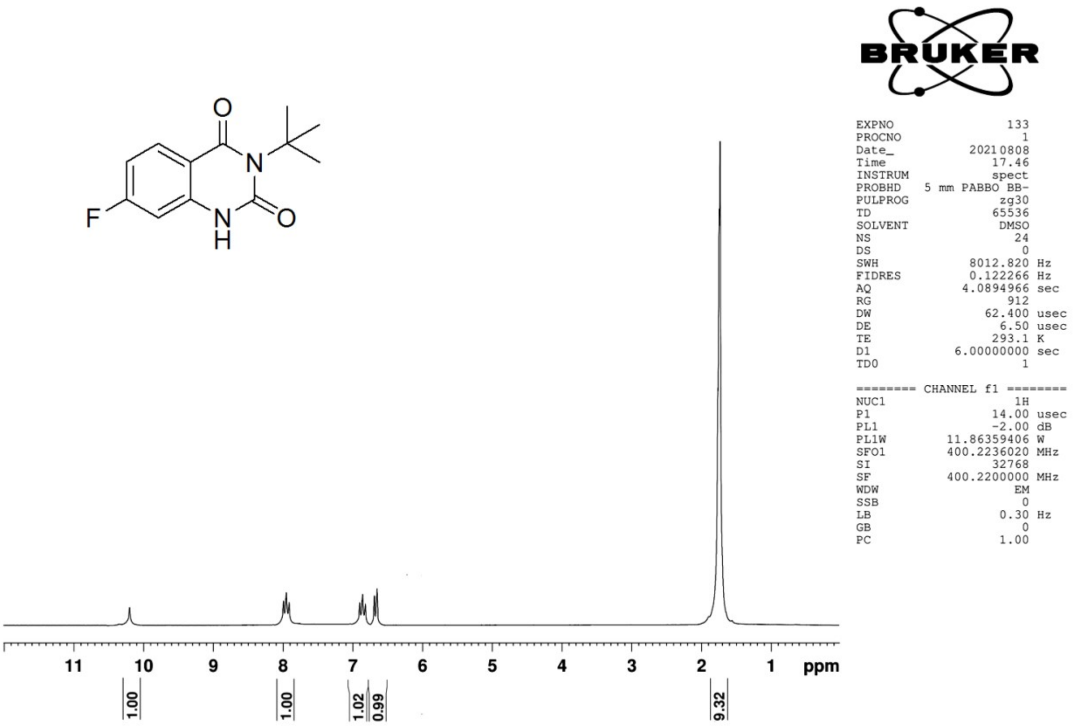

F
N
H
O
N
O
BRUKER
EXPNO 133
PROCNO 1
Date_ 20210808
Time 17.46
INSTRUM spect
PROBHD 5 mm PABBO BB-
PULPROG zg30
TD 65536
SOLVENT DMSO
NS 24
DS 0
SWH 8012.820 Hz
FIDRES 0.122266 Hz
AQ 4.0894966 sec
RG 912
DW 62.400 usec
DE 6.50 usec
TE 293.1 K
D1 6.00000000 sec
TD0 1
======== CHANNEL f1 ========
NUC1 1H
P1 14.00 usec
PL1 -2.00 dB
PL1W 11.86359406 W
SFO1 400.2236020 MHz
SI 32768
SF 400.2200000 MHz
WDW EM
SSB 0
LB 0.30 Hz
GB 0
PC 1.00
11 10 9 8 7 6 5 4 3 2 1 ppm
1.00
1.00
1.02
0.99
9.32

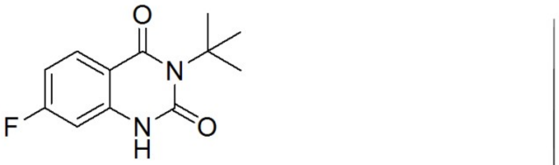

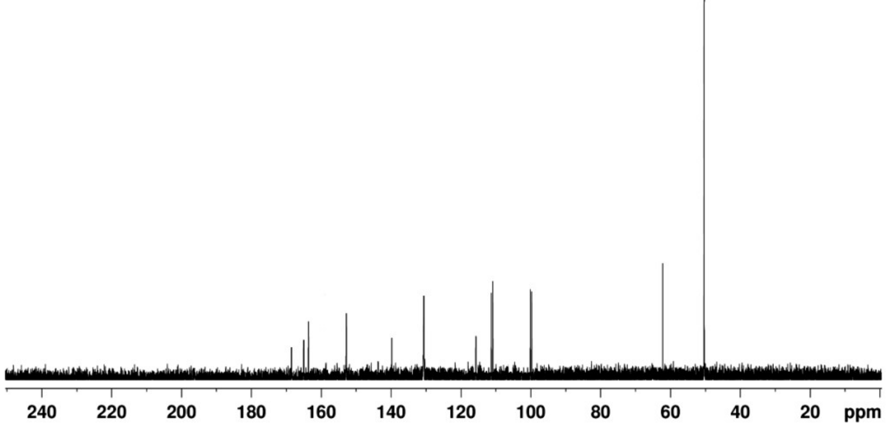

EXPNO	134	
PROCNO	1	
Date_	20210808	
Time	17.51	
INSTRUM	spect	
PROBHD	5 mm PABBO BB-	
PULPROG	zgpg	
TD	65536	
SOLVENT	DMSO	
NS	110	
DS	0	
SWH	25252.525	Hz
FIDRES	0.385323	Hz
AQ	1.2976629	sec
RG	2050	
DW	19.800	usec
DE	6.50	usec
TE	293.3	K
D1	3.00000000	sec
D11	0.03000000	sec
TD0	1	
======== CHANNEL f1 ========		
NUC1	13C	
P1	9.00	usec
PL1	-0.90	dB
PL1W	42.02801895	W
SFO1	100.6479784	MHz
======== CHANNEL f2 ========		
CPDPRG2	waltz16	
NUC2	1H	
PCPD2	90.00	usec
PL2	-2.00	dB
PL12	14.16	dB
PL13	17.90	dB
PL2W	11.86359406	W
PL12W	0.28722104	W
PL13W	0.12139934	W
SFO2	400.2216009	MHz
SI	32768	
SF	100.6353990	MHz
WDW	EM	
SSB	0	
LB	1.00	Hz
GB	0	
PC	1.40	

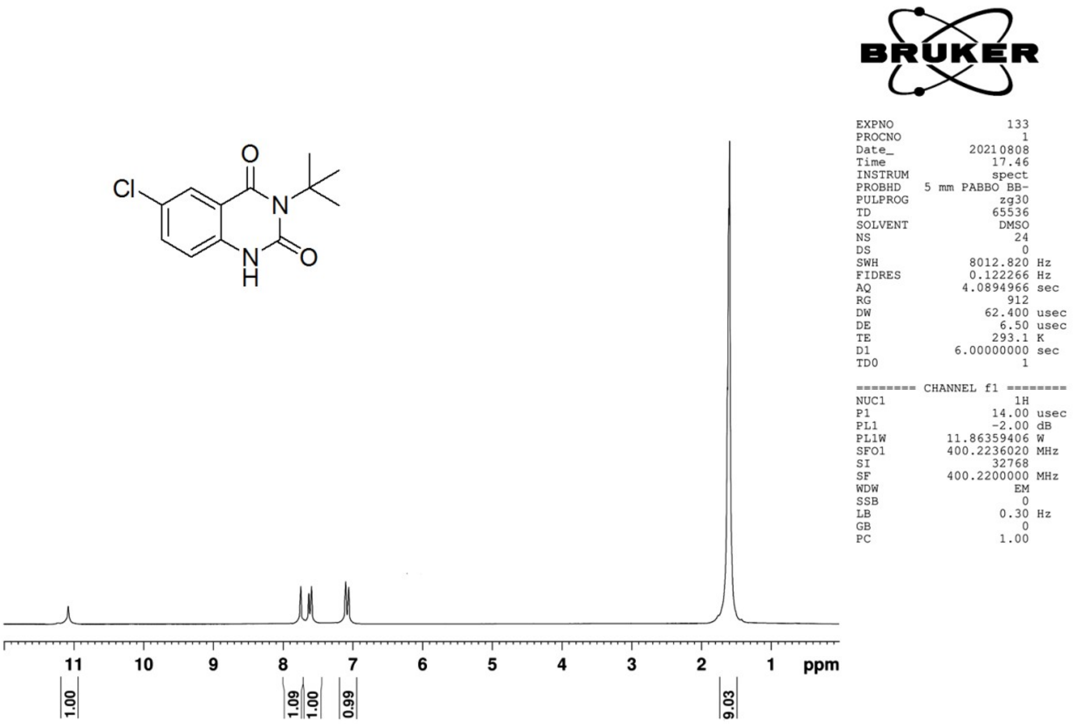

Cl
O
N
N
H
O
BRUKER
EXPNO 133
PROCNO 1
Date_ 20210808
Time 17.46
INSTRUM spect
PROBHD 5 mm PABBO BB-
PULPROG zg30
TD 65536
SOLVENT DMSO
NS 24
DS 0
SWH 8012.820 Hz
FIDRES 0.122266 Hz
AQ 4.0894966 sec
RG 912
DW 62.400 usec
DE 6.50 usec
TE 293.1 K
D1 6.00000000 sec
TD0 1
======== CHANNEL f1 ========
NUC1 1H
P1 14.00 usec
PL1 -2.00 dB
PL1W 11.86359406 W
SFO1 400.2236020 MHz
SI 32768
SF 400.2200000 MHz
WDW EM
SSB 0
LB 0.30 Hz
GB 0
PC 1.00
11 10 9 8 7 6 5 4 3 2 1 ppm
1.00
1.09
1.00
0.99
9.03

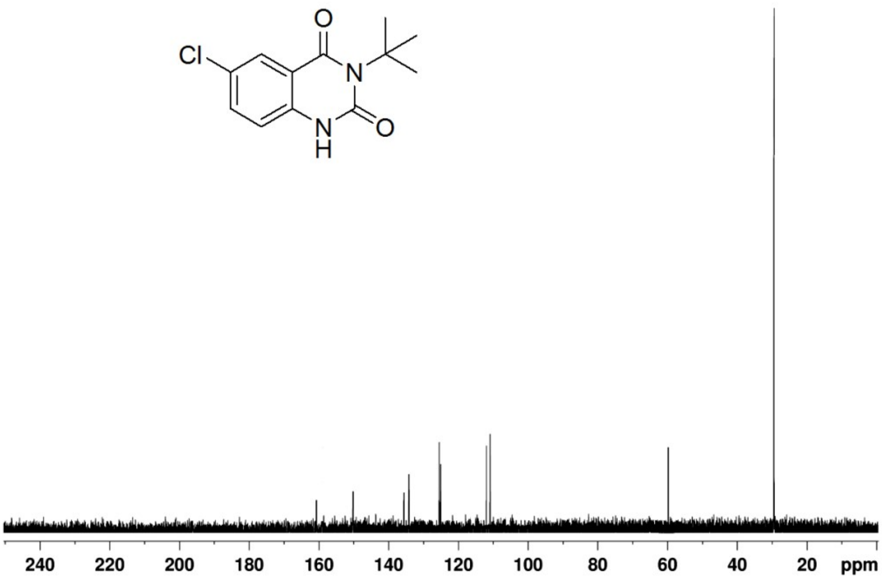


```
EXPNO              134
PROCNO               1
Date_         20210808
Time             12.36
INSTRUM          spect
PROBHD   5 mm PABBO BB-
PULPROG           zgpg
TD               65536
SOLVENT           DMSO
NS                 110
DS                   0
SWH          25252.525 Hz
FIDRES        0.385323 Hz
AQ           1.2976629 sec
RG                2050
DW              19.800 usec
DE                6.50 usec
TE               293.3 K
D1          3.00000000 sec
D11         0.03000000 sec
TD0                  1

======== CHANNEL f1 ========
NUC1               13C
P1                9.00 usec
PL1              -0.90 dB
PL1W       42.02801895 W
SFO1       100.6479784 MHz

======== CHANNEL f2 ========
CPDPRG2        waltz16
NUC2                1H
PCPD2            90.00 usec
PL2              -2.00 dB
PL12             14.16 dB
PL13             17.90 dB
PL2W       11.86359406 W
PL12W       0.28722104 W
PL13W       0.12139934 W
SFO2       400.2216009 MHz
SI               32768
SF         100.6353990 MHz
WDW                 EM
SSB                  0
LB                1.00 Hz
GB                   0
PC                1.40
```
